# A simple model for eletrocommunication – “refractoriness avoidance response”?

**DOI:** 10.1186/1471-2202-15-S1-P68

**Published:** 2014-07-21

**Authors:** Rafael T Guariento, Thiago S Mosqueiro, Angel A Caputi, Reynaldo D Pinto

**Affiliations:** 1 Instituto de Física de São Carlos, Universidade de São Paulo, São Carlos, SP 13566-590, Brazil; 2 Department of Integrative and Computational Neurosciences, Instituto de Investigaciones Biológicas Clemente Estable, Montevideo, Uruguai

## 

Weakly field electric fishes have an electric sense with two simultaneously processed tasks, called electrolocation and electrocommunication [1]. In pulse-type electric fishes, the first task is perceived by deformations of the self-generated electric field [2], and the later one by the precise timestamp of its own pulses and those generated by its conspecifics. Some observed changes in the timestamps of two communicating fish are different from those observed during the so called “Jamming Avoidance Response” of wave-type electric fishes [3]. Although in some fish there actually is a rate separation mechanism (coincidence avoidance), in most fish these rate changes are only transient [3] and has been reproduced using recursive models [4,5]. However it is well known that the electrosensory path of the fish is stimulated by the discharge of its conspecifics during the interval between two self-generated discharges (stimulus phase) [6]. Moreover, second order neurons responsible for the fish's self-detection show very long refractory period of about 10ms due to the activation of a low threshold K^+^ conductance [7]. This has led to hypothesis’ that there is not a coincidence avoidance effect, but instead an attempt to avoid firing just after its conspecific. Thus, a fish sensory processed signal is not jammed by a conspecific signal [6]. Agreeing with that, there is evidence that the fish adjusts its own signal to make the other one fire at a “preferential” phase [8], and this is probably related to dominance [3]. These effects can be observed in an integrate-and-fire model with non-linear inputs taking into account a phase preference for eliciting acceleration [9]. As the refractoriness of the fast electrosensory path is the most important jamming effect we call this effect “electrosensory refractoriness avoidance response” (RAR). Here, we extend this idea and test it using a simplified model that take this effects into account. We propose a model with two integrate-and-fire neurons per fish and a non-linear feedback loop between them. The first neuron represents the fish's pulse, and the other one a “sensory neuron”. The feedback is non-linear and has the effect of increase the frequency of firing. This model has been implemented both in an electronic hardware and in a computer simulation and allows us using it to mimic a conspecific during electro-communication encounters with real fish.
